# *N*-Benzyl-2-(2-chloro-4-methyl­phen­oxy)acetamide

**DOI:** 10.1107/S1600536808022526

**Published:** 2008-07-26

**Authors:** Zhu-Bo Li, Hua Zuo, Wen-Liang Dong, Xiao-Yan He, Zhang-Bao Chen

**Affiliations:** a College of Pharmaceutical Sciences, Southwest University, Chongqing 400716, People’s Republic of China; b Shandong University of Traditional Chinese Medicine, Jinan 250355, People’s Republic of China

## Abstract

The structure determination of the title compound, C_16_H_16_ClNO_2_, was performed as part of a project on the inter­actions between small organic mol­ecules and proteins. In the crystal structure, the dihedral angle between the two aromatic rings is 16.14 (12)°. The molecules are connected via N—H⋯O hydrogen bonding into chains, which extend in the direction of the *b* axis.
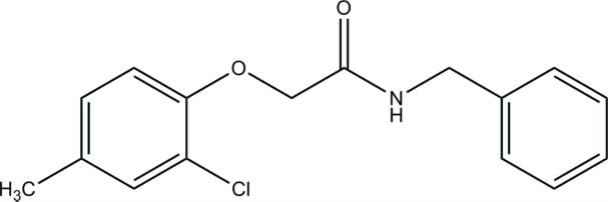

## Experimental

### 

#### Crystal data

C_16_H_16_ClNO_2_
*M* *_r_* = 289.75
Orthorhombic, 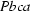

*a* = 11.9900 (18) Å
*b* = 9.2986 (14) Å
*c* = 25.868 (4) Å
*V* = 2884.1 (7) Å^3^
*Z* = 8
Mo *K*α radiation
μ = 0.27 mm^−1^
*T* = 273 (2) K
0.15 × 0.10 × 0.10 mm

#### Data collection

Bruker APEXII CCD area-detector diffractometer
Absorption correction: none
15831 measured reflections
3286 independent reflections
1964 reflections with *I* > 2σ(*I*)
*R*_int_ = 0.051

#### Refinement

*R*[*F* ^2^ > 2σ(*F* ^2^)] = 0.045
*wR*(*F* ^2^) = 0.133
*S* = 0.96
3286 reflections
185 parameters
H atoms treated by a mixture of independent and constrained refinement
Δρ_max_ = 0.21 e Å^−3^
Δρ_min_ = −0.22 e Å^−3^

### 

Data collection: *APEX2* (Bruker, 2005 ▶); cell refinement: *APEX2*; data reduction: *APEX2*; program(s) used to solve structure: *SIR97* (Altomare *et al.*, 1999 ▶); program(s) used to refine structure: *SHELXL97* (Sheldrick, 2008 ▶); molecular graphics: *SHELXTL* (Sheldrick, 2008 ▶); software used to prepare material for publication: *WinGX* (Farrugia, 1999 ▶).

## Supplementary Material

Crystal structure: contains datablocks I, global. DOI: 10.1107/S1600536808022526/nc2110sup1.cif

Structure factors: contains datablocks I. DOI: 10.1107/S1600536808022526/nc2110Isup2.hkl

Additional supplementary materials:  crystallographic information; 3D view; checkCIF report

## Figures and Tables

**Table 1:** Hydrogen-bond geometry (Å, °)

*D*—H⋯*A*	*D*—H	H⋯*A*	*D*⋯*A*	*D*—H⋯*A*
N—H⋯O2^i^	0.80 (2)	2.09 (3)	2.885 (2)	169 (2)

Symmetry code: (i) 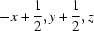.

**Fig. 1.**
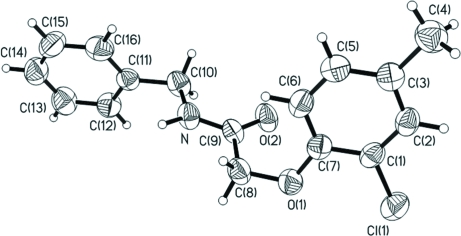
The molecular structure of (I) with labelling and displacement ellipsoids drawn at the 50% probability level.

## supplementary crystallographic information

### Experimental

A solution of 2-chloro-4-methylphenol (1.0 mmol), *N*-benzyl-2-chloroacetamide (1.1 mmol), K_2_CO_3_ (1.1 mmol) in CH_3_CN (20 ml) was refluxed for 3 h. Afterwards the mixture has cooled down to room temperature the solvent was evaporated under reduced pressure. The residue was poured into water and adjusted the pH 6–7. The aqueous phase was extracted with ethyl acetate, washed with brine and dried over anhydrous MgSO_4_. Finally the product was purified by column chromatography on silica gel. Crystals of (I) suitable for X-ray diffraction were obtained by cooling of a solution of the title compound in a mixture of ethylacetate and hexane.

### Refinement

All C-H atoms were placed in geometrically calculated positions and refined using a riding model with C—H = 0.97 Å (for CH_2 _groups) and 0.96 Å (for CH_3_ groups), their isotropic displacement parameters were set to 1.2 times (1.5 times for CH_3_ groups) the equivalent displacement parameter of their parent atoms. The N-H H atom was freely refined.

### Crystal data

**Table:**

C_16_H_16_ClNO_2_	*D*_x_ = 1.335 Mg m^−^^3^
*M**_r_* = 289.75	Mo *K*α radiation λ = 0.71073 Å
Orthorhombic, *P**b**c**a*	Cell parameters from 2043 reflections
*a* = 11.9900 (18) Å	θ = 2.9–22.9º
*b* = 9.2986 (14) Å	µ = 0.27 mm^−^^1^
*c* = 25.868 (4) Å	*T* = 273 (2) K
*V* = 2884.1 (7) Å^3^	Block, colorless
*Z* = 8	0.15 × 0.10 × 0.10 mm
*F*_000_ = 1216	

### Data collection

**Table:**

Bruker SMART CCD area-detector diffractometer	1964 reflections with *I* > 2σ(*I*)
Radiation source: fine-focus sealed tube	*R*_int_ = 0.051
Monochromator: graphite	θ_max_ = 27.5º
*T* = 273(2) K	θ_min_ = 1.6º
φ and ω scans	*h* = −15→15
Absorption correction: none	*k* = −12→11
15831 measured reflections	*l* = −25→33
3286 independent reflections	

### Refinement

**Table:**

Refinement on *F*^2^	Secondary atom site location: difference Fourier map
Least-squares matrix: full	Hydrogen site location: inferred from neighbouring sites
*R*[*F*^2^ > 2σ(*F*^2^)] = 0.046	H atoms treated by a mixture of independent and constrained refinement
*wR*(*F*^2^) = 0.133	*w* = 1/[σ^2^(*F*_o_^2^) + (0.06*P*)^2^ + 0.774*P*] where *P* = (*F*_o_^2^ + 2*F*_c_^2^)/3
*S* = 0.96	(Δ/σ)_max_ < 0.001
3286 reflections	Δρ_max_ = 0.21 e Å^−^^3^
185 parameters	Δρ_min_ = −0.22 e Å^−^^3^
Primary atom site location: structure-invariant direct methods	Extinction correction: none

### Special details

**Table:**

Geometry. All e.s.d.'s (except the e.s.d. in the dihedral angle between two l.s. planes) are estimated using the full covariance matrix. The cell e.s.d.'s are taken into account individually in the estimation of e.s.d.'s in distances, angles and torsion angles; correlations between e.s.d.'s in cell parameters are only used when they are defined by crystal symmetry. An approximate (isotropic) treatment of cell e.s.d.'s is used for estimating e.s.d.'s involving l.s. planes.
Refinement. Refinement of *F*^2^ against ALL reflections. The weighted *R*-factor *wR* and goodness of fit *S* are based on *F*^2^, conventional *R*-factors *R* are based on *F*, with *F* set to zero for negative *F*^2^. The threshold expression of *F*^2^ > σ(*F*^2^) is used only for calculating *R*-factors(gt) *etc*. and is not relevant to the choice of reflections for refinement. *R*-factors based on *F*^2^ are statistically about twice as large as those based on *F*, and *R*- factors based on ALL data will be even larger.

### Fractional atomic coordinates and isotropic or equivalent isotropic displacement parameters (Å^2^)

**Table:**

	*x*	*y*	*z*	*U*_iso_*/*U*_eq_	
N	0.25818 (17)	0.4733 (2)	0.38688 (7)	0.0475 (5)	
H	0.2699 (18)	0.557 (3)	0.3929 (9)	0.049 (7)*	
Cl1	0.03007 (5)	0.17559 (7)	0.57574 (3)	0.0657 (2)	
O1	0.12962 (13)	0.38711 (16)	0.50994 (6)	0.0518 (4)	
O2	0.19800 (14)	0.26379 (16)	0.42037 (6)	0.0557 (4)	
C1	0.17217 (18)	0.2116 (2)	0.57204 (8)	0.0449 (5)	
C2	0.24535 (19)	0.1359 (2)	0.60276 (8)	0.0479 (5)	
H2	0.2178	0.0675	0.6257	0.057*	
C3	0.35942 (19)	0.1599 (2)	0.60016 (9)	0.0484 (5)	
C4	0.4390 (2)	0.0750 (3)	0.63326 (10)	0.0669 (7)	
H4A	0.5140	0.1053	0.6263	0.100*	
H4B	0.4315	−0.0255	0.6255	0.100*	
H4C	0.4221	0.0911	0.6691	0.100*	
C5	0.3965 (2)	0.2623 (3)	0.56529 (9)	0.0518 (6)	
H5	0.4726	0.2803	0.5626	0.062*	
C6	0.32366 (19)	0.3384 (2)	0.53445 (9)	0.0498 (6)	
H6	0.3514	0.4062	0.5113	0.060*	
C7	0.20977 (18)	0.3153 (2)	0.53747 (8)	0.0431 (5)	
C8	0.1658 (2)	0.4792 (2)	0.46959 (8)	0.0510 (6)	
H8A	0.2239	0.5422	0.4825	0.061*	
H8B	0.1038	0.5387	0.4584	0.061*	
C9	0.21020 (18)	0.3948 (2)	0.42362 (8)	0.0428 (5)	
C10	0.2954 (2)	0.4080 (2)	0.33864 (8)	0.0510 (6)	
H10A	0.2365	0.3473	0.3250	0.061*	
H10B	0.3597	0.3477	0.3454	0.061*	
C11	0.32561 (18)	0.5196 (2)	0.29900 (8)	0.0441 (5)	
C12	0.2495 (2)	0.5609 (2)	0.26193 (9)	0.0547 (6)	
H12	0.1787	0.5201	0.2619	0.066*	
C13	0.2765 (2)	0.6617 (3)	0.22488 (10)	0.0653 (7)	
H13	0.2241	0.6880	0.2001	0.078*	
C14	0.3801 (2)	0.7230 (3)	0.22441 (10)	0.0655 (7)	
H14	0.3985	0.7901	0.1991	0.079*	
C15	0.4562 (2)	0.6855 (3)	0.26101 (12)	0.0686 (7)	
H15	0.5263	0.7282	0.2611	0.082*	
C16	0.4294 (2)	0.5832 (3)	0.29846 (10)	0.0608 (7)	
H16	0.4820	0.5577	0.3232	0.073*	

### Atomic displacement parameters (Å^2^)

**Table:**

	*U*^11^	*U*^22^	*U*^33^	*U*^12^	*U*^13^	*U*^23^
N	0.0699 (13)	0.0305 (10)	0.0421 (10)	−0.0025 (9)	0.0022 (9)	−0.0059 (8)
Cl1	0.0507 (4)	0.0739 (5)	0.0724 (5)	−0.0075 (3)	0.0088 (3)	0.0085 (3)
O1	0.0589 (10)	0.0498 (9)	0.0468 (9)	0.0051 (8)	0.0059 (7)	0.0039 (7)
O2	0.0827 (12)	0.0299 (8)	0.0546 (10)	0.0008 (8)	0.0042 (8)	−0.0021 (7)
C1	0.0479 (12)	0.0466 (12)	0.0403 (12)	−0.0045 (10)	0.0084 (10)	−0.0056 (10)
C2	0.0601 (14)	0.0441 (12)	0.0393 (11)	−0.0043 (11)	0.0071 (11)	−0.0013 (10)
C3	0.0573 (14)	0.0465 (13)	0.0413 (12)	0.0001 (11)	0.0005 (10)	−0.0075 (10)
C4	0.0665 (16)	0.0714 (17)	0.0627 (16)	0.0036 (14)	−0.0056 (13)	0.0011 (14)
C5	0.0480 (13)	0.0502 (14)	0.0572 (14)	−0.0033 (11)	0.0017 (11)	−0.0088 (11)
C6	0.0588 (14)	0.0441 (12)	0.0465 (13)	−0.0063 (11)	0.0104 (11)	−0.0021 (10)
C7	0.0525 (13)	0.0409 (11)	0.0360 (11)	−0.0006 (10)	0.0058 (9)	−0.0072 (9)
C8	0.0690 (15)	0.0373 (11)	0.0468 (13)	0.0048 (11)	0.0035 (11)	0.0006 (10)
C9	0.0546 (13)	0.0341 (11)	0.0397 (12)	0.0046 (9)	−0.0050 (10)	0.0012 (9)
C10	0.0690 (15)	0.0424 (12)	0.0414 (12)	0.0060 (11)	−0.0006 (11)	−0.0038 (10)
C11	0.0501 (13)	0.0410 (11)	0.0410 (12)	0.0054 (10)	0.0032 (10)	−0.0050 (9)
C12	0.0540 (14)	0.0569 (14)	0.0531 (14)	−0.0011 (12)	−0.0045 (11)	0.0017 (11)
C13	0.0795 (19)	0.0671 (16)	0.0494 (14)	0.0046 (14)	−0.0054 (13)	0.0077 (13)
C14	0.084 (2)	0.0584 (16)	0.0541 (16)	0.0035 (14)	0.0201 (15)	0.0046 (12)
C15	0.0574 (16)	0.0654 (17)	0.083 (2)	−0.0106 (13)	0.0191 (14)	−0.0030 (15)
C16	0.0523 (14)	0.0636 (16)	0.0665 (17)	0.0048 (12)	−0.0075 (12)	−0.0037 (13)

### Geometric parameters (Å, °)

**Table:**

N—C9	1.329 (3)	C6—H6	0.9300
N—C10	1.458 (3)	C8—C9	1.521 (3)
N—H	0.80 (2)	C8—H8A	0.9700
Cl1—C1	1.739 (2)	C8—H8B	0.9700
O1—C7	1.370 (3)	C10—C11	1.503 (3)
O1—C8	1.418 (3)	C10—H10A	0.9700
O2—C9	1.230 (2)	C10—H10B	0.9700
C1—C2	1.377 (3)	C11—C16	1.378 (3)
C1—C7	1.390 (3)	C11—C12	1.378 (3)
C2—C3	1.387 (3)	C12—C13	1.380 (3)
C2—H2	0.9300	C12—H12	0.9300
C3—C5	1.385 (3)	C13—C14	1.366 (4)
C3—C4	1.505 (3)	C13—H13	0.9300
C4—H4A	0.9600	C14—C15	1.361 (4)
C4—H4B	0.9600	C14—H14	0.9300
C4—H4C	0.9600	C15—C16	1.395 (4)
C5—C6	1.378 (3)	C15—H15	0.9300
C5—H5	0.9300	C16—H16	0.9300
C6—C7	1.385 (3)		
			
C9—N—C10	121.07 (19)	O1—C8—H8B	109.3
C9—N—H	117.9 (16)	C9—C8—H8B	109.3
C10—N—H	120.9 (16)	H8A—C8—H8B	107.9
C7—O1—C8	117.56 (17)	O2—C9—N	123.1 (2)
C2—C1—C7	121.3 (2)	O2—C9—C8	121.51 (19)
C2—C1—Cl1	119.61 (17)	N—C9—C8	115.30 (18)
C7—C1—Cl1	119.12 (17)	N—C10—C11	111.74 (17)
C1—C2—C3	121.2 (2)	N—C10—H10A	109.3
C1—C2—H2	119.4	C11—C10—H10A	109.3
C3—C2—H2	119.4	N—C10—H10B	109.3
C2—C3—C5	117.3 (2)	C11—C10—H10B	109.3
C2—C3—C4	120.9 (2)	H10A—C10—H10B	107.9
C5—C3—C4	121.8 (2)	C16—C11—C12	118.1 (2)
C3—C4—H4A	109.5	C16—C11—C10	121.4 (2)
C3—C4—H4B	109.5	C12—C11—C10	120.5 (2)
H4A—C4—H4B	109.5	C11—C12—C13	121.1 (2)
C3—C4—H4C	109.5	C11—C12—H12	119.4
H4A—C4—H4C	109.5	C13—C12—H12	119.4
H4B—C4—H4C	109.5	C14—C13—C12	120.2 (3)
C6—C5—C3	121.8 (2)	C14—C13—H13	119.9
C6—C5—H5	119.1	C12—C13—H13	119.9
C3—C5—H5	119.1	C15—C14—C13	119.8 (2)
C5—C6—C7	120.8 (2)	C15—C14—H14	120.1
C5—C6—H6	119.6	C13—C14—H14	120.1
C7—C6—H6	119.6	C14—C15—C16	120.2 (2)
O1—C7—C6	125.9 (2)	C14—C15—H15	119.9
O1—C7—C1	116.42 (19)	C16—C15—H15	119.9
C6—C7—C1	117.6 (2)	C11—C16—C15	120.5 (2)
O1—C8—C9	111.79 (17)	C11—C16—H16	119.8
O1—C8—H8A	109.3	C15—C16—H16	119.8
C9—C8—H8A	109.3		
			
C7—C1—C2—C3	−0.3 (3)	C10—N—C9—O2	2.8 (3)
Cl1—C1—C2—C3	178.82 (16)	C10—N—C9—C8	−174.4 (2)
C1—C2—C3—C5	−0.3 (3)	O1—C8—C9—O2	10.6 (3)
C1—C2—C3—C4	−179.0 (2)	O1—C8—C9—N	−172.11 (19)
C2—C3—C5—C6	0.3 (3)	C9—N—C10—C11	168.6 (2)
C4—C3—C5—C6	179.0 (2)	N—C10—C11—C16	83.7 (3)
C3—C5—C6—C7	0.3 (3)	N—C10—C11—C12	−96.4 (2)
C8—O1—C7—C6	9.4 (3)	C16—C11—C12—C13	0.8 (3)
C8—O1—C7—C1	−171.68 (17)	C10—C11—C12—C13	−179.1 (2)
C5—C6—C7—O1	178.02 (19)	C11—C12—C13—C14	−0.1 (4)
C5—C6—C7—C1	−0.9 (3)	C12—C13—C14—C15	−0.8 (4)
C2—C1—C7—O1	−178.11 (18)	C13—C14—C15—C16	1.0 (4)
Cl1—C1—C7—O1	2.7 (2)	C12—C11—C16—C15	−0.5 (3)
C2—C1—C7—C6	0.9 (3)	C10—C11—C16—C15	179.4 (2)
Cl1—C1—C7—C6	−178.22 (16)	C14—C15—C16—C11	−0.4 (4)
C7—O1—C8—C9	71.4 (2)		

### Hydrogen-bond geometry (Å, °)

**Table:**

*D*—H···*A*	*D*—H	H···*A*	*D*···*A*	*D*—H···*A*
N—H···O2^i^	0.80 (2)	2.09 (3)	2.885 (2)	169 (2)

Symmetry codes: (i) −*x*+1/2, *y*+1/2, *z*.
